# Supplementary material for: Multi‐dataset identification of innovative feature genes and molecular mechanisms in keratoconus
Source: J Cell Mol Med. 2024 Sep 19;28(18):e70079. doi: 10.1111/jcmm.70079 (PMC11412914; doi:10.1111/jcmm.70079)
Supplement: Supplementary file 2 — Material S2. [file JCMM-28-e70079-s002.zip › 7.GSEA analysis/5.RNF217-AS1/KEGG_PYRIMIDINE_METABOLISM.html]

Details for gene set KEGG\_PYRIMIDINE\_METABOLISM[GSEA]

|  || Dataset | RNF217-AS1.group.cls#h\_versus\_l |
| Phenotype | group.cls#h\_versus\_l |
| Upregulated in class | l |
| GeneSet | KEGG\_PYRIMIDINE\_METABOLISM |
| Enrichment Score (ES) | -0.32793623 |
| Normalized Enrichment Score (NES) | -1.4496028 |
| Nominal p-value | 0.13034624 |
| FDR q-value | 0.30215633 |
| FWER p-Value | 0.825 |
Table: GSEA Results Summary

  

Fig 1: Enrichment plot: KEGG\_PYRIMIDINE\_METABOLISM      
 Profile of the Running ES Score & Positions of GeneSet Members on the Rank Ordered List

  

| SYMBOL | TITLE | RANK IN GENE LIST | RANK METRIC SCORE | RUNNING ES | CORE ENRICHMENT || 1 | UPP2 | na | 1406 | 0.401 | 0.0007 | No |
| 2 | NT5E | na | 1485 | 0.394 | 0.0390 | No |
| 3 | NT5C1B | na | 2371 | 0.323 | 0.0468 | No |
| 4 | UPB1 | na | 4405 | 0.237 | 0.0125 | No |
| 5 | UPP1 | na | 6611 | 0.164 | -0.0342 | No |
| 6 | NT5C2 | na | 6735 | 0.161 | -0.0212 | No |
| 7 | POLR1A | na | 8068 | 0.126 | -0.0467 | No |
| 8 | ENTPD8 | na | 8746 | 0.111 | -0.0548 | No |
| 9 | DPYS | na | 9307 | 0.099 | -0.0608 | No |
| 10 | POLR2H | na | 10375 | 0.080 | -0.0834 | No |
| 11 | POLE | na | 10398 | 0.080 | -0.0758 | No |
| 12 | NUDT2 | na | 10655 | 0.075 | -0.0754 | No |
| 13 | NT5C | na | 13158 | 0.040 | -0.1436 | No |
| 14 | UPRT | na | 14449 | 0.025 | -0.1782 | No |
| 15 | TYMP | na | 14474 | 0.025 | -0.1763 | No |
| 16 | ENTPD6 | na | 14646 | 0.023 | -0.1789 | No |
| 17 | CMPK1 | na | 14760 | 0.022 | -0.1799 | No |
| 18 | POLR2J3 | na | 14815 | 0.021 | -0.1793 | No |
| 19 | POLD4 | na | 14819 | 0.021 | -0.1772 | No |
| 20 | POLR2J | na | 14839 | 0.021 | -0.1756 | No |
| 21 | CTPS2 | na | 15268 | 0.016 | -0.1863 | No |
| 22 | ENTPD5 | na | 15383 | 0.015 | -0.1880 | No |
| 23 | CANT1 | na | 15894 | 0.010 | -0.2017 | No |
| 24 | POLR2C | na | 16068 | 0.008 | -0.2059 | No |
| 25 | ENTPD4 | na | 16251 | 0.006 | -0.2105 | No |
| 26 | PRIM2 | na | 16280 | 0.006 | -0.2107 | No |
| 27 | POLD1 | na | 16368 | 0.005 | -0.2127 | No |
| 28 | POLR1E | na | 16630 | 0.002 | -0.2200 | No |
| 29 | POLR1D | na | 16828 | 0.000 | -0.2256 | No |
| 30 | ITPA | na | 16864 | -0.000 | -0.2266 | No |
| 31 | PNP | na | 16875 | -0.000 | -0.2269 | No |
| 32 | POLR3D | na | 16907 | -0.001 | -0.2277 | No |
| 33 | POLR2A | na | 16956 | -0.001 | -0.2290 | No |
| 34 | AK3 | na | 18017 | -0.011 | -0.2585 | No |
| 35 | TXNRD1 | na | 18026 | -0.011 | -0.2576 | No |
| 36 | POLE4 | na | 18097 | -0.012 | -0.2584 | No |
| 37 | POLR2I | na | 18217 | -0.013 | -0.2605 | No |
| 38 | POLR2J2 | na | 18415 | -0.015 | -0.2647 | No |
| 39 | NME3 | na | 18738 | -0.018 | -0.2722 | No |
| 40 | DUT | na | 18799 | -0.018 | -0.2720 | No |
| 41 | UMPS | na | 19067 | -0.021 | -0.2776 | No |
| 42 | POLR2L | na | 19097 | -0.021 | -0.2763 | No |
| 43 | NME1 | na | 19169 | -0.022 | -0.2761 | No |
| 44 | POLR3B | na | 19405 | -0.024 | -0.2804 | No |
| 45 | POLR3A | na | 19531 | -0.025 | -0.2814 | No |
| 46 | POLR2F | na | 19959 | -0.030 | -0.2906 | No |
| 47 | UCK1 | na | 20107 | -0.031 | -0.2917 | No |
| 48 | UCKL1 | na | 20280 | -0.033 | -0.2933 | No |
| 49 | POLR2G | na | 20302 | -0.033 | -0.2905 | No |
| 50 | NME2 | na | 20629 | -0.036 | -0.2962 | No |
| 51 | NME5 | na | 21730 | -0.048 | -0.3230 | Yes |
| 52 | POLD3 | na | 21754 | -0.048 | -0.3188 | Yes |
| 53 | POLR1C | na | 21908 | -0.050 | -0.3180 | Yes |
| 54 | POLR2E | na | 22042 | -0.051 | -0.3166 | Yes |
| 55 | CDA | na | 22365 | -0.055 | -0.3203 | Yes |
| 56 | POLE3 | na | 22462 | -0.056 | -0.3173 | Yes |
| 57 | DTYMK | na | 22578 | -0.057 | -0.3148 | Yes |
| 58 | NT5C3A | na | 22939 | -0.061 | -0.3189 | Yes |
| 59 | POLD2 | na | 23076 | -0.063 | -0.3164 | Yes |
| 60 | POLR3H | na | 23306 | -0.066 | -0.3162 | Yes |
| 61 | POLA1 | na | 23390 | -0.067 | -0.3117 | Yes |
| 62 | POLR2D | na | 23397 | -0.067 | -0.3050 | Yes |
| 63 | RRM1 | na | 23408 | -0.067 | -0.2984 | Yes |
| 64 | DHODH | na | 23456 | -0.068 | -0.2928 | Yes |
| 65 | PNPT1 | na | 23526 | -0.069 | -0.2878 | Yes |
| 66 | TK1 | na | 23790 | -0.072 | -0.2879 | Yes |
| 67 | POLR2B | na | 24796 | -0.087 | -0.3080 | Yes |
| 68 | NME6 | na | 24886 | -0.089 | -0.3014 | Yes |
| 69 | UCK2 | na | 24949 | -0.089 | -0.2940 | Yes |
| 70 | POLR3GL | na | 25131 | -0.092 | -0.2897 | Yes |
| 71 | CTPS1 | na | 25792 | -0.103 | -0.2981 | Yes |
| 72 | POLR2K | na | 25798 | -0.103 | -0.2876 | Yes |
| 73 | POLR3K | na | 26033 | -0.108 | -0.2833 | Yes |
| 74 | POLR3G | na | 26041 | -0.108 | -0.2724 | Yes |
| 75 | ENTPD3 | na | 27247 | -0.131 | -0.2937 | Yes |
| 76 | DCTD | na | 27267 | -0.131 | -0.2807 | Yes |
| 77 | NME7 | na | 28109 | -0.151 | -0.2894 | Yes |
| 78 | NT5C1A | na | 28564 | -0.164 | -0.2856 | Yes |
| 79 | POLR1B | na | 29029 | -0.177 | -0.2808 | Yes |
| 80 | DCK | na | 29232 | -0.183 | -0.2678 | Yes |
| 81 | ENTPD1 | na | 29277 | -0.184 | -0.2501 | Yes |
| 82 | TXNRD2 | na | 29295 | -0.185 | -0.2316 | Yes |
| 83 | PRIM1 | na | 29707 | -0.197 | -0.2232 | Yes |
| 84 | TK2 | na | 29905 | -0.203 | -0.2080 | Yes |
| 85 | CAD | na | 30639 | -0.228 | -0.2056 | Yes |
| 86 | NME4 | na | 30750 | -0.232 | -0.1849 | Yes |
| 87 | POLR3F | na | 30818 | -0.234 | -0.1627 | Yes |
| 88 | POLA2 | na | 30950 | -0.240 | -0.1417 | Yes |
| 89 | NT5M | na | 31015 | -0.243 | -0.1186 | Yes |
| 90 | RRM2B | na | 31129 | -0.247 | -0.0964 | Yes |
| 91 | TYMS | na | 31610 | -0.269 | -0.0825 | Yes |
| 92 | POLE2 | na | 32955 | -0.357 | -0.0846 | Yes |
| 93 | DPYD | na | 33263 | -0.381 | -0.0542 | Yes |
| 94 | RRM2 | na | 33544 | -0.411 | -0.0200 | Yes |
| 95 | CMPK2 | na | 34197 | -0.525 | 0.0153 | Yes |
Table: GSEA details [plain text format]

  

Fig 2: KEGG\_PYRIMIDINE\_METABOLISM      
 Blue-Pink O' Gram in the Space of the Analyzed GeneSet

  

Fig 3: KEGG\_PYRIMIDINE\_METABOLISM: Random ES distribution      
 Gene set null distribution of ES for **KEGG\_PYRIMIDINE\_METABOLISM**
